# Definition of a prospective payment system to reimburse emergency departments

**DOI:** 10.1186/1472-6963-13-409

**Published:** 2013-10-11

**Authors:** Rosella Levaggi, Marcello Montefiori

**Affiliations:** 1 Department of Economics and Management, University of Brescia, Via Faustino, 74, Brescia 25122, Italy; 2 Department of Economics, University of Genoa, Via Vivaldi, 5, Genova 16126, Italy

**Keywords:** Emergency department, Prospective payment system, Healthcare services

## Abstract

**Background:**

Payers are increasingly turning to Prospective Payment Systems (PPSs) because they incentivize efficiency, but their application to emergency departments (EDs) is difficult because of the high level of uncertainty and variability in the cost of treating each patient.

To the best of our knowledge, our work represents the first attempt at defining a PPS for this part of hospital activity.

**Methods:**

Data were specifically collected for this study and relate to 1011 patients who were triaged at an ED of a major Italian hospital, during 1 week in December 2010.

The cost for each patient was analytically estimated by adding up several components: 1) physician and other staff costs that were imputed on the basis of the time each physician claimed to have spent treating the patient; 2) the cost for each test/treatment each patient actually underwent; 3) overhead costs, shared among patients using the time elapsed between first examination and discharge from the ED.

**Results:**

The distribution of costs by triage code shows that, although the average cost increases across the four triage groups, the variance within each code is quite high. The maximum cost for a yellow code is €1074.7, compared with €680 for red, the most serious code. Using cluster analysis, the red code cluster is enveloped by yellow, and their costs are therefore indistinguishable, while green codes span all cost groups. This suggests that triage code alone is not a good proxy for the patient cost, and that other cost drivers need to be included.

**Conclusions:**

Crude triage codes cannot be used to define PPSs because they are not sufficiently correlated with costs and are characterized by large variances. However, if combined with other information, such as the number of laboratory and non-laboratory tests/examinations, it is possible to define cost groups that are sufficiently homogeneous to be reimbursed prospectively. This should discourage strategic behavior and allow the ED to break even or create profits, which can be reinvested to improve services. The study provides health policy administrators with a new and feasible tool to implement prospective payment for EDs, and improve planning and cost control.

## Background

Emergency departments (EDs) are responsible for a large share of overall hospitalization and diagnostic activity, but little research exists on their cost and their impact on healthcare expenditure [1]. ED treatments are patient specific and the variance in cost of each case is fairly high and difficult to predict. For this reason, most healthcare systems use retrospective systems to reimburse their activity [2,3]. However, cost reimbursement has several drawbacks: it does not allow the costs of EDs to be controlled and it may allow hospitals to 'play’ strategically [4,5].

In a context where hospital admissions are paid for using Prospective Payment Systems (PPSs), providers can shift costs to the purchaser by timing the admission of patients from ED to another hospital ward. Alternatively, patients may undergo diagnostic tests in the ED that should have been routinely performed after admission to a ward, and would not then have been reimbursed separately.

For this reason, some authors have proposed the use of a PPS for EDs, but the high level of uncertainty and variability in the resources needed to treat each patient [6] has discouraged most of them from pursuing this objective. Despite these difficulties, there are countries who are experimenting with new solutions to reimburse the activity of their EDs. For instance, Australia has recently proposed a Diagnosis-Related Group (DRG) based system [7] and Belgium finances part of its ED activity prospectively [8,9].

EDs generally use triage codes for priority setting that are related to a patient’s level of severity (clinical need). At an international level, there are several different triage systems. One common system, used for instance in the United States, is the Emergency Severity Index (ESI) where patients are assigned to one of five different severity groups, according to the observation of different variables such as vital functions, life or organ threat, and expected resource use (e.g., x-rays, laboratory tests/examinations, and consultations) [10]. ESI-1 refers to the most urgent patients and ESI-5 to the least.

In this work, we will refer to a different algorithm for prioritizing patients, which is used by many EDs in Europe. It is based on four color codes that measure to what extent the patient’s condition is critical. Each code has a color tag: red codes (the most urgent patients) must be attended to immediately, for yellow and green codes some waiting is possible, while white codes (the least urgent) represent an inappropriate use of the ED. Several studies have been proposed to test the reliability of the triage system in defining the actual medical urgency of patients [11] and its ability to predict hospitalizations or mortality [12]. Other contributions focus on the ability of triage codes to predict actual resource use in terms, for instance, length of stay, hospitalization, or x ray [10,13,14].

The present paper differs from the existing literature because it starts from an analytical cost allocation of the actual costs incurred by hospitals when treating patients in an ED. The individual cost is then used to propose a PPS for an ED. To the best of our knowledge, our work represents the first attempt to define a PPS for this important part of hospital activity.

## Methods

### Study design

One of the most important shortcomings of previous studies is their inability to determine the actual cost for each patient accessing an ED. In this study, we have tried to overcome this problem using a unique database that matches patient information with data specifically collected for this purpose on the intensity and the cost of care. In this way, we can determine the cost of each access to the ED, and use this information to define a PPS scheme for ED patients. The first indicator we tested was the triage code. Patients were sorted according to their triage code, then for each group and with reference to all the clinical and cost variables of interest, the average and the confidence intervals were computed. We subsequently clustered patients, by a k-means procedure, into 20 different classes of total cost to identify the cost drivers of ED activity. This enabled us to determine whether triage codes could be used to define a PPS scheme, or whether additional cost drivers needed to be included, and if so, which, in order to develop a suitable algorithm for reimbursement for patients.

The cluster analysis showed that triage codes alone were not a good candidate, but that the number of laboratory and non-laboratory tests and examinations was a key cost driver. Our algorithm therefore combines the triage code with the number of laboratory and non-laboratory tests/examinations. We have grouped patients into three uniform cost classes (A, B, and C) that represent the classification of our proposed PPS. Our classification starts from the triage codes. All white codes are assigned to class A while all yellow and red codes, which are indistinguishable from a cost point of view, according to the cluster analysis, are assigned to group C. Green codes are split among the three groups using the number of non-laboratory and laboratory tests/examinations as a discriminant. Any green code patients who required less than or equal to the average number of examinations prescribed to white codes were assigned to group A. Those green code patients whose total cost was, because of the number of non-laboratory tests/examinations, at least as high as the cost of the upper triage group (yellow/red) here grouped into category C. This procedure then allows us to identify a set of green code patients, homogeneous in terms of costs, who represent group B.

The reimbursement for patients in the different groups was set to discourage any strategic behavior by the hospital either in terms of upcoding (e.g., from green to yellow) or downgrading (e.g., from yellow to green).

Finally to test for the reliability of our approach, we split our sample into two equal parts using the date of admittance as the discriminant and evaluated the difference between the actual cost incurred to treat the patients and the reimbursement the hospital would have received with our PPS policy.

### Ethical considerations

The study was carried out as part of routine checks conducted in the ED of the hospital and so ethical approval was not required. As is the case with all studies conducted in the hospital environment, the management of the hospital approved the study protocol. The management is responsible for ensuring the ethical aspects of all hospital activities. Furthermore, the entire study was organized in conjunction with ED teams. On entering the hospital, all patients sign an informed consent form regarding treatment in the hospital and the terms and conditions of any treatment. Finally, the research was carried out in full accordance with Italian law on privacy (Decreto Legislativo 30 giugno 2003, n. 196).

### Data analysis

Data refer to patients who were triaged at the ED of E.O. Ospedali Galliera in Genoa, Italy, during a sample week from Thursday 9th December 2010, 8:00 pm until Thursday 16th December 2010, 8:00 pm. For each patient, data on personal characteristics, clinical pathway, and costs were specifically collected for this project. The electronic data processing center of the ED records the following information:

i. Date and time of arrival

ii. Medical attendant (the identification code of the member of staff dealing with the incident)

iii. Triage entry code

iv. Patient’s personal information (in particular, gender and date of birth)

v. Date and time of first examination

vi. Number and type of laboratory and non-laboratory tests/examinations

vii. Patient outcome

viii. Date and hour of discharge (patient report closing time)

This information is not sufficient to allocate medical and staff costs, which represent a very large component (around 35%) of the total ED costs [6,15-17]. The time elapsing between arrival at ED and discharge (which can be obtained from each patient file) is not a good approximation of the time required to treat the patient because of the two-level system to access treatment. On arrival, the patient is seen by nurses who assign a specific triage code ranging from white (inappropriate access) to red (emergency), which determines the priority for attention from the medical staff. For this reason, white and green code patients may wait for a long time, especially when more severe cases are being treated. For the same reasons, the time elapsing from the first examination (which represents the moment in which the patient is taken into care by the ED) and the time the patient is discharged, cannot be properly used to allocate medical and staff costs. In fact, patients may have to stay inside the ED for “observation” due to their health condition, but this stay does not necessarily require physicians’ care.

To provide a reliable and analytical imputation of the time devoted to each patient by physicians, physicians were asked to make a contemporaneous (at the point at which the patient file was closed) report of the actual time they spent treating each patient. At the end of each shift, the team of researchers checked and collected the data, asking physicians to fill in any missing data before they left the ED. Information concerning diagnostic tests and other medical treatments was provided by the hospital electronic data processing center. The economic cost for each test/treatment (such as laboratory tests, non-laboratory tests and examinations, and x-ray tests) that each patient actually underwent was then analytically imputed [18].

The cost per patient consists of three main cost components:

physician and other staff costs

patient-specific direct costs (diagnostic tests, x-ray tests, laboratory tests, examinations, other)

ED overhead costs (cleaning, mortgages, kitchen and laundry, medical devices, other)

The cost per patient was thus estimated via a 'bottom-up’ approach [19] by adding up the three different components. All cost data used in the study are in 2010 euros.

#### Physicians and other staff costs

For physicians and other staff costs, an hourly wage was obtained from the department’s annual balance sheet, which was then multiplied by the amount of time each physician stated he/she had devoted to the patient.

#### Patient specific direct costs

The number and the type of tests were provided by the hospital electronic data processing center and linked to the other information concerning each patient by the 'ID entrance code’. The price for laboratory and non-laboratory tests was determined using the E.O. Galliera Hospital internal price list^a^; if this information was not available, the corresponding regional tariff was used.

#### ED overhead costs

Overhead costs represent 14% of the total ED costs. From the ED annual balance sheet, we obtained an estimate of overhead costs for a sample week. The total cost was then shared among patients, using the time elapsed between first examination and discharge from ED [20], assuming a positive correlation between overhead costs and time spent in the ED. In fact, we believe that this is a reliable proxy of a patient’s specific consumption of overhead ED costs, provided that the patient, during his stay, occupies a bed and absorbs ED resources including cleaning, kitchen and laundry, and medical devices.

## Results

Table 1 summarizes the most important characteristics of our sample.

**Table 1:** Sample dataset - descriptive statistics and cost composition by triage code

	***Triage code***				
	**White**	**Green**	**Yellow**	**Red**	**All**
***Obs***	81*(8.01%)*	689*(68.15%)*	219*(21.66%)*	22*(2.18%)*	1011*(100%)*
**Bio & Clinical variables (mean values)**					
Age	37.83	45.64	64.13	76.18	49.68
	*[33.91 – 41.74]*^*a*^	*[44.11 - 47.17]*	*[61.24 - 67.01]*	*[68.36 - 84.00]*	*[48.30 - 51.06]*
Gender (female)	0.64	0.50	0.52	0.45	0.51
	*[0.54 - 0.75]*	*[0.46 - 0.53]*	*[0.45 - 0.59]*	*[0.23 - 0.68]*	*[0.48 - 0.54]*
Time elapsing between 1st exam. and exit	0.52	1.90	4.49	2.18	2.35
	*[-0.04 - 1.07]*	*[1.70 - 2.10]*	*[3.76 - 5.22]*	*[1.55 - 2.80]*	*[2.13 - 2.54]*
N. of non-lab. prescr..	1.90	3.14	5.08	6.95	3.54
	*[1.77 - 2.03]*	*[3.02 - 3.26]*	*[4.72 - 5.43]*	*[5.96 - 7.95]*	*[3.41 - 3.67]*
N.of lab. prescr.	0.09	0.38	1.14	1.14	0.54
	*[0.01 - 0.16]*	*[0.33 - 0.43]*	*[1.03 - 1.26]*	*[0.98 - 1.29]*	*[0.49 - 0.59]*
N. of lab tests	0.49	3.42	11.88	13.36	5.23
	*[-0.04 - 1.03]*	*[2.97 - 3.87]*	*[10.99 - 12.76]*	*[11.22 - 15.51]*	*[4.80 - 5.67]*
Lab tests per each lab prescr.	5.71	8.96	10.40	11.76	9.71
Time devoted to patients	3.96	7.84	14.49	19.23	9.22
	*[2.58 - 5.34]*	*[7.39 - 8.29]*	*[13.26 - 15.73]*	*[11.26 - 27.19]*	*[8.73 - 9.72]*
**Cost variables (mean values)**					
Total cost	87.83	189.38	340.54	407.39	218.73
	*[77.08 - 98.58]*	*[181.98 - 196.77]*	*[320.83 - 360.24]*	*[350.41 - 464.37]*	*[210.48 - 226.98]*
Medical Doctors	21.02	35.00	59.14	68.80	39.84
	*[19.75 - 22.30]*	*[34.24 - 35.77]*	*[57.55 - 60.73]*	*[64.73 - 72.87]*	*[38.87 - 40.82]*
Nurses- other Personnel- Admin Staff.	19.17	32.89	55.46	63.96	37.36
	*[17.98 - 20.35]*	*[32.16 - 33.62]*	*[53.97 - 56.96]*	*[60.14 - 67.78]*	*[36.43 - 38.28]*
Mortgages. Kitchen & Laundry. Clean & other exp.	1.45	5.66	12.86	6.69	6.91
	*[-0.02 - 2.92]*	*[5.13 - 6.21]*	*[10.93 - 14.81]*	*[4.77 - 8.62]*	*[6.31 - 7.52]*
Health services. Surg. & Med. devices. Drugs	0.42	3.07	4.93	4.38	3.29
	*[0.13 - 0.73]*	*[2.87 - 3.27]*	*[4.44 - 5.42]*	*[3.12 - 5.64]*	*[3.10 - 3.48]*
X-ray	8.26	50.77	97.90	103.85	58.72
	*[0.32 - 16.21]*	*[46.06 - 55.48]*	*[83.13 - 112.67]*	*[55.61 - 152.10]*	*[53.82 - 63.64]*
Non-Lab tests	33.61	34.41	50.60	99.01	39.26
	*[30.86 - 36.37]*	*[33.06 - 35.77]*	*[47.12 - 54.09]*	*[75.32 - 122.70]*	*[37.79 - 40.73]*
Lab tests	1.14	8.02	28.27	32.85	12.40
	*[-0.04 - 2.32]*	*[6.99 - 9.07]*	*[26.09 - 30.46]*	*[27.39 - 38.31]*	*[11.37 - 13.44]*

^*a*^*95% confidence intervals for means.*

About 68% of patients accessed the ED with a green triage code, 22% had a yellow code, and only a small fraction had a red code.

Patients with more serious triage codes had a higher average age and there were slightly more women than men in the white code group. The time between examination and discharge increased with increasing severity across the code groups, as expected for the first three codes, although red code patients did not seem to require significantly more time than green code patients.

For non-laboratory prescriptions, there was a one-to-one correspondence with the tests/examinations actually performed, although this is not the case for laboratory tests, because they may be prescribed in batches. For this reason, the number of laboratory prescriptions also represents a poor proxy for the actual number of tests performed. From Table 1, it is possible to see that the average number of both laboratory and non-laboratory tests/examinations increases with the severity of the triage code, as does the average number of laboratory tests/examinations per prescription.

**Figure 1:**
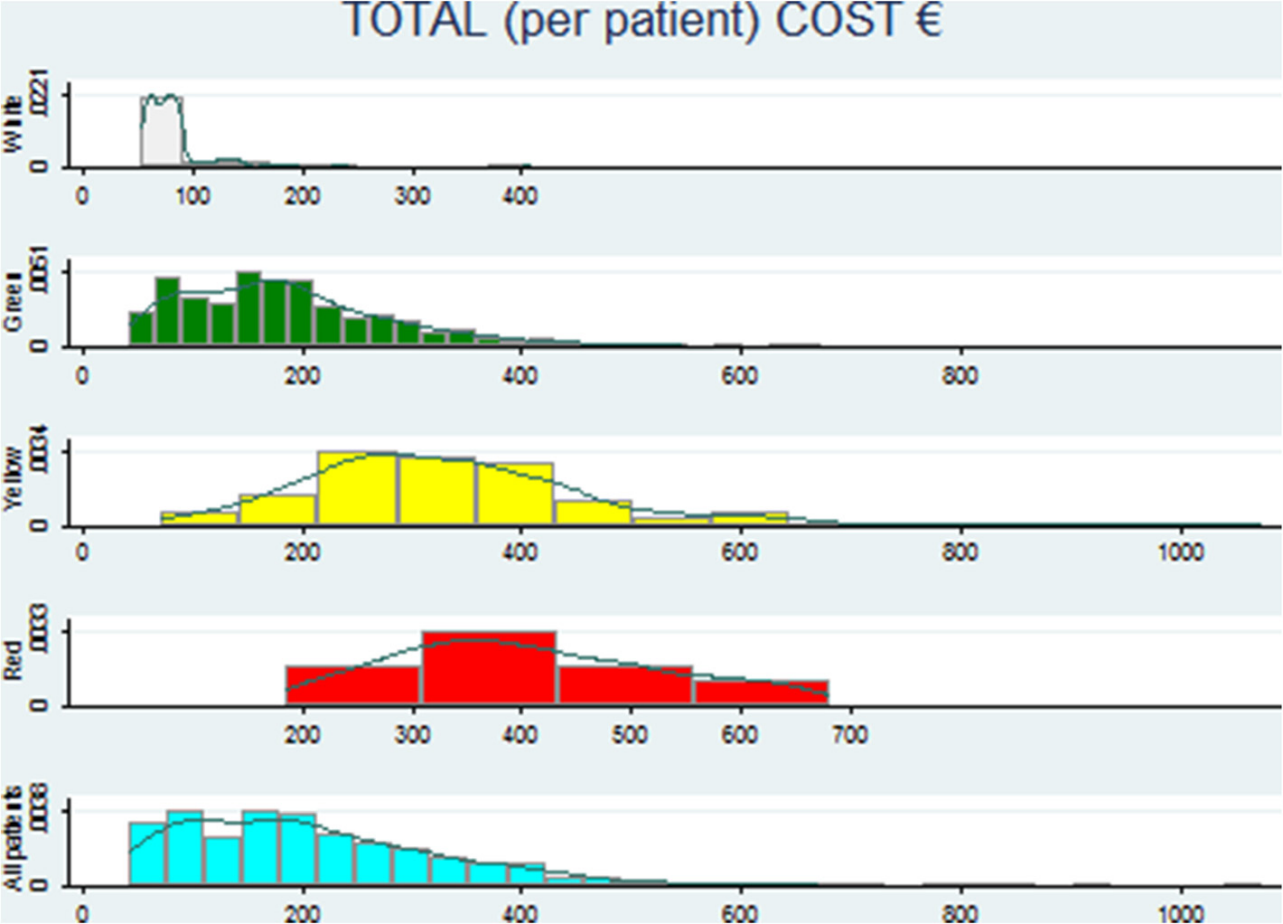
Distribution of total cost per patient within each triage code.

The distribution of costs by triage code (Figure 1) shows that although the average cost increases with severity of coding, the variance within each code is high, thus suggesting that the triage code alone may not be a good proxy of the cost per patient. It is also interesting that the most expensive patients are yellow codes rather than red, and that the cost of red codes is always within the range of variation for yellow codes. In other words, yellow and red codes are indistinguishable from a cost perspective.

**Figure 2:**
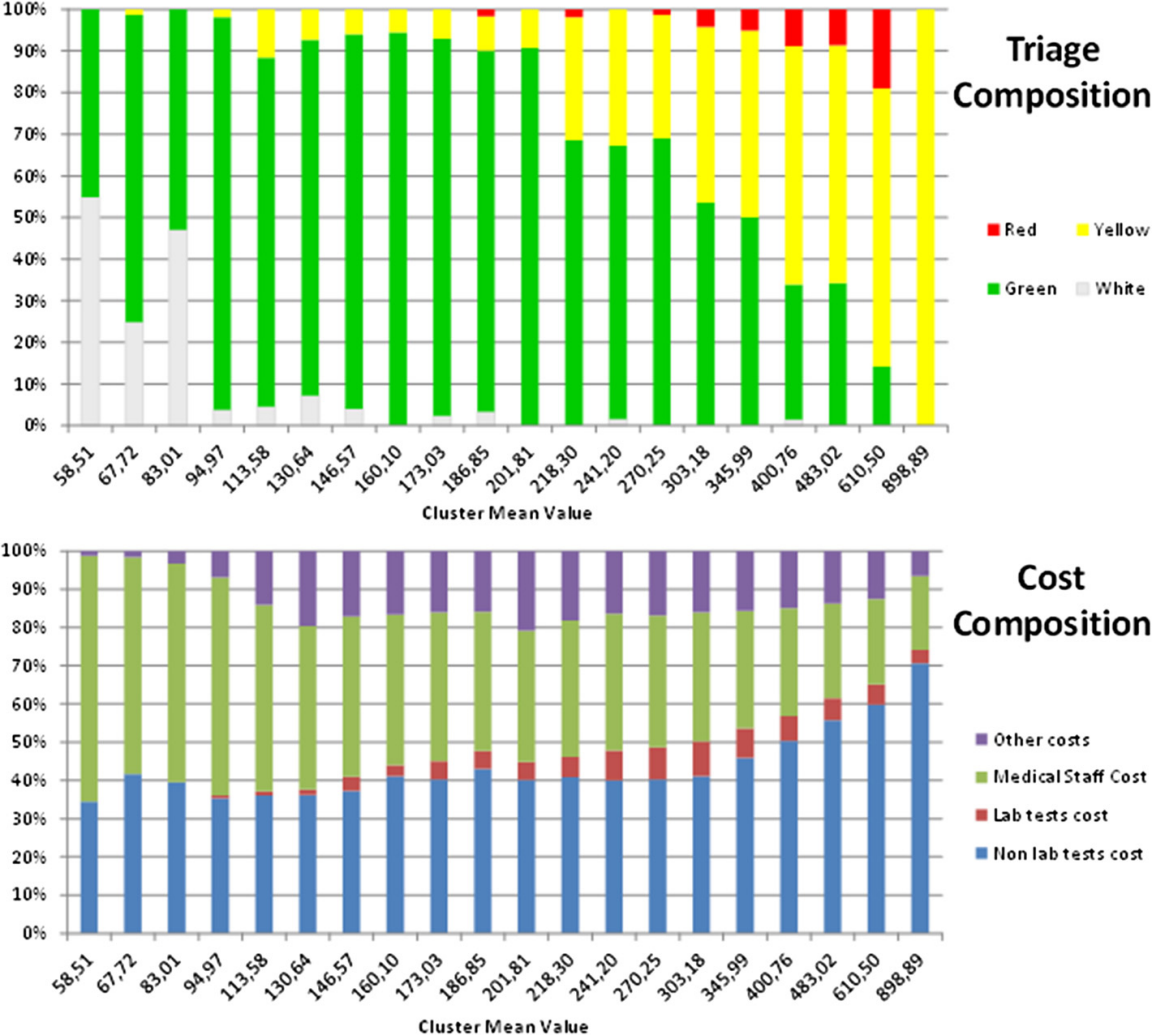
Cluster analysis: Triage vs. cost composition.

The main results of the cluster analysis are presented in Figure 2. White codes are clustered in the first cost classes while green codes span almost all. Red codes are enveloped by yellow ones. That is, from a cost point of view, they do not seem to have any distinguishing characteristics.

Non laboratory tests/examinations emerge as the real cost driver for ED patients. White codes require an examination and, in general, no laboratory tests and are clustered in the first cost classes. As the number of non-laboratory examinations/tests increases, so does total cost and the tests comprise an increasing share of that total. On the basis of these results, we can conclude that a combination of triage codes and the number of tests performed can be used to design different categories/groups of ED access, forming the basis of a PPS.

From Table 1, we can see that the average number of non-laboratory prescriptions for white codes is below two, with a 95% confidence interval. On average, the number of non-laboratory prescriptions for green codes is much higher (3.14). Moreover, almost all white codes (93% of the total) do not require any laboratory tests (the average number of laboratory prescriptions per white code patient is 0.09). For this reason, all green code patients with fewer than two non-laboratory prescriptions and no laboratory tests/examinations were grouped with white codes.

Similarly, green code patients with seven or more non-laboratory tests/examinations, that is, the average number for red codes, were included in group C. The economic analysis shows that the distinction between green and yellow/red codes mainly depends on the number of tests that have been prescribed. The average cost for a red/yellow code is €346.6. The average cost for green codes with six non-laboratory tests or examinations is €343.5, for seven is €361, and for eight is €403. That means that they have a cost well within the yellow/red range. This allows us to define the following three groups, each of which is homogeneous from the cost point of view.

### Group A

This first group can be considered as 'inappropriate access’. It pools white codes with green codes requiring no laboratory examinations and a maximum of two non-laboratory examinations.

### Group B

The second group consists of green code patients who required more than two, but fewer than seven non-laboratory examinations or at least one laboratory test/examination.

### Group C

The third group comprises green code patients requiring at least seven non-laboratory examinations, and all yellow and red codes.

Group A contained 291 patients (out of 1011). The actual per patient average cost was €94, and we suggest that the reimbursement level set for group A patients should be the same. Attendance is considered inappropriate for all white code patients (by definition) and any green code patients with no laboratory tests/examinations and a maximum of two non-laboratory tests/examinations.

The proposed PPS policy is summarized in Table 2.

**Table 2:** Prospective payment system

	**Description**	**N.Obs**	**Actual mean cost**	**Total**	**PPS**	**Total**	**Diff.**	**% Diff**
Group A	White & Green inappropriate	291	94	27354	**94**	27354	0	0,00%
Group B	Green with n. of non-lab tests/examinations <7	449	218	97882	**222**	99678	1796	1.83%
Group C	Green (with n. of non-lab tests/examinations *≥*7) + Yellow & Red	271	353	95663	**347**	94037	1626	-1.69%
**Tot.**		**1011**		**220899**		**221069**	**170**	**0,07%**

Group B included 449 green code patients. This group was characterized by an actual average cost of €218, but the PPS suggested is €222. This means that for each patient in this group the hospital would gain on average €4.

Finally, group C contained 271 patients. The payment we propose for this group is €347, i.e., a reimbursement below the actual average cost of €353 that the hospital incurs when treating these patients. In fact, the PPS is set equal to the average per patient cost for yellow and red code patients. Green code patients who receive more than seven non-laboratory tests/examinations cost on average more than €347 but they are, deliberately, underestimated.

## Discussion

The analysis presented in this paper is one of the first attempts of which we are aware to study the relationship between triage codes and the cost of treating patients in ED. The data usually available through patients’ files are not sufficient for the analysis, but we were able to use a unique dataset that matches patient information with data specifically collected for this purpose, relating to the intensity and cost of care.

As a first step towards the definition of a PPS, patients were grouped according to their triage code and data summarized in Table 1. A first interesting result emerges: the time elapsing between examination and discharge increases with the severity code for the first three codes (white, green, and yellow), but counter intuitively, red codes seem to be 'faster’ than yellow, and do not seem to require more time then green. This result may be explained in several ways: red codes are usually very critical patients who may be admitted more quickly to other departments and yellow codes may include patients who need to be monitored for a longer period than red codes. Finally, it may well be that, from a cost point of view, there is no difference between these two. This intuition seems to be confirmed by data on the time devoted by medical staff to each patient.

Looking at the number of prescriptions for non-laboratory and laboratory tests/examinations, we note that whereas the former increases, as expected, with the triage code, the latter, surprisingly, does not. This odd result is actually easily explainable. For non-laboratory prescriptions, there is a one-to-one correspondence with the tests/examinations performed, while laboratory tests are prescribed in batches, hence the number of prescriptions is a poor proxy for the actual number of tests/examinations performed. This is confirmed by the fact that the average number of tests/examinations per prescription (see Table 1) increases, as it might be expected, with the triage code.

Figure 1 provides evidence that crude triage codes alone cannot be used to design a PPS because of the high variance within groups. The design of a PPS requires a set of observable variables, possibly outside the provider’s control, which allow the definition of homogeneous groups of patients in terms of cost. If the triage code was a good indicator, costs per triage code should have a relatively small variance within each group, whereas the mean of the cost of each triage code should be significantly different. Several statistical tests can be performed to check for these characteristics, but they often imply that the distribution of the observations is not significantly different from a normal distribution, a characteristic that our data do not possess, as shown in Figure 1. The triage code is a system for priority setting in EDs that should reflect the severity of a patient’s condition, and not his/her need for care. Their use for pricing ED care is therefore controversial.

A further investigation on this issue was made possible through the identification of 20 clusters. Each cluster groups patients who are homogeneous in terms of their total cost. The cluster analysis by cost composition is presented in Figure 2. White codes are different in their cost from yellow/red, while green over-run both categories. Lower cost classes (prevalence of white and green codes) mainly require an examination and some tests. This result may be interpreted in terms of appropriateness and upcoding. White codes (inappropriate use of ED) do not need care from this department: an examination and some reassurance on health status is all that is required. In Italy, access to ED is free except for white codes, where a €25 fee is charged. As a consequence, it may well be possible that in some cases, nurses upcode patients from white to green, so as to deliver faster and free treatment.

For higher cost classes, the main cost driver seems to be non-laboratory tests/examinations. This may well be explained by the fact that critical patients usually require treatment from specialists outside the ED and the use of more sophisticated diagnostic techniques raises the share of non-laboratory costs. This information can be used to design a PPS using triage codes, together with the number of laboratory and non-laboratory tests/examinations as a discriminant. Furthermore, analysis of Figure 2 seems to suggest that yellow and red codes are not statistically different and, in terms of reimbursement, could be pooled together. Using this information, it was possible to define three different categories (Groups A, B, and C) of ED access that could be reimbursed using a PPS.

Group A encompasses inappropriate patients: all the white code patients and those green code patients requiring no laboratory tests/examinations and fewer than two non-laboratory tests/examinations. Green code patients with these characteristics are indistinguishable, from a clinical or cost point of view, from white code patients. In fact, looking at Table 1, it is noticeable that the average number of non-laboratory tests for white code patients is less than two (with a 95% confidence interval) and almost all of the white code patients (93%) do not even require a laboratory test/examination. In other words, as set out earlier, we believe that these green patients might have been upcoded.

For groups B and C, it was necessary to set a reimbursement that would give the right incentives to contain costs and limit upcoding from green to red/yellow. We aimed to shift reimbursement from high- to low-cost patients by setting a tariff for group B that was slightly higher than the average observed cost.

Upcoding from white to green (see Table 2) is partially discouraged because less complicated green code patients are reimbursed at the same level as white code patients. However, upcoding from A to B may still occur, especially for patients who are quite close to the threshold. Upcoding from group B to C is certainly not expedient: the threshold is very high and the additional reimbursement is well below the actual cost incurred. Finally, it should be noted that, to balance the accounts, the reimbursement for group B is actually higher than the cost incurred. Green codes with fewer than seven non-laboratory tests/examinations have an actual average cost of €218 per patient, but we propose a reimbursement of €222.

This result is well in line with suggestions in the agency literature. In the presence of asymmetry of information and unseen actions, it is in fact optimal to increase the reimbursement for the best-case scenario (green code patient, not complicated) and reduce the payment for the worst state (green code patient, complicated). In this way, hospitals have no interest in upcoding (increasing the number of non-laboratory tests/examinations) but instead, receive the extra profit assigned to group B patients when they are able to contain the number of tests/examinations and, by this behavior, get a more profitable reimbursement.

In our system, most green codes (which are also the largest group of patients admitted to the department) are in the B group, which is generously reimbursed and for whom a reduction in the number of tests/examinations can lead to an increase in hospital profits. From this perspective, our system should offer an ED enough incentive to ensure the appropriate use of testing.

Nonetheless, our policy may induce hospitals to increase the number of prescribed treatments for 'borderline’ green code patients [21]. A shift from group A to group B would give hospitals a substantial increase in reimbursement. In our sample, there were 162 'borderline’ green code patients, defined as patients who, in the sample week, required no laboratory tests and two non-laboratory tests/examinations, i.e., about 16% of the total number of patients. To show the possible effects of upcoding, we carried out a simulation where all these patients were upcoded. In this case, the hospital would increase its income by about 9%. This would be the worst scenario. To upcode patients it is necessary to inflate non-laboratory tests/examinations, i.e., specialist consultations and x-rays. This may be very difficult to justify and can be easily detected, and is therefore deemed unlikely to happen very often in practice.

Finally, to test the reliability of our approach, the sample was split into two equal parts using the date of admittance as the discriminant. In so doing, we were interested in evaluating the difference between the actual cost incurred to treat patients and the reimbursement the hospital would have received with the new reimbursement policy (Table 3).

**Table 3:** Simulations for PPS

**1st sample**							
	**N.Obs**	**Actual mean cost**	**Total**	**PPS**	**Total**	**Diff.**	**% Diff**
Group A	146	91.51	13359.91	**94**	13724	364.09	2.73%
Group B	224	216.58	48515.02	**222**	49728	1212.98	2.5%
Group C	135	358.07	48340.07	**347**	46845	-1495.07	-3.09%
**Tot.**	**505**		**110215.00**		**110297**	**82.00**	**0.07%**
**2nd sample**							
	**N.Obs**	**Actual mean cost**	**Total**	**PPS**	**Total**	**Diff.**	**% Diff**
Group A	145	96.72	14024.07	**94**	13630	-394.07	-2.81%
Group B	225	219.81	49457.14	**222**	49950	492.86	0.99%
Group C	136	348.82	47439.52	**347**	47192	-247.52	-0.52%
**Tot.**	**506**		**110920.73**		**110772**	**-148.73**	**-0.13%**

Our system allows the difference between actual cost and reimbursement to be kept to a minimum. The reimbursement proposed overestimates the cost of the first sample and underestimates the second because, by construction, it allows accounts to be balanced over the whole period. What is relevant is by how little the proposed PPS under- and overestimates the actual cost. In the first period, the difference is 0.07% and in the second -0.13%. This implies that our system is quite robust in terms of predicting the actual cost of EDs.

### Limitations

The present work uses data from a single hospital and for a limited period of time. This may reduce the robustness of the results obtained.

A second limitation concerns the criteria used to distribute some of the indirect costs. For instance, the time elapsing between first examination and discharge is used to attribute the share of overhead ED costs to each patient. The rationale for this choice is that patients, during their stay, occupy a bed and absorb ED resources. However, it is possible that the time elapsed may not in fact be an accurate proxy for use of resources, for example, if some overheads are not time-dependent, or do not depend on the number of patients present.

A further limitation concerns the fact that asking staff to self-report time devoted to patients introduces an unavoidable bias in the computations. Several techniques could be adopted to reduce this problem (see, for example, [16]) by a sort of standardization of the data. However, standardization presents the drawback of artificially rescaling the actual time declared. In other words, some information may be lost. For this reason we have chosen to retain the original data.

Last but not least, it is important to note that any prospective payment system that includes the number/volume of tests to determine the amount of payment is vulnerable to the unintended consequence of incentivizing unnecessary or avoidable testing to move a visit from one level of payment to a higher one. This would negate the expected efficiency gains, which are the main reason why PPS are implemented.

## Conclusions

Most healthcare systems use retrospective reimbursement systems for EDs, which may not be efficient for several reasons. A PPS would solve some of the problems, but the high level of uncertainty and the variability in the cost of resources needed to treat each patient is a serious hurdle to the use of such a payment system. The aim of this study was to provide an accurate estimate of the actual cost the hospital incurs when treating patients in the ED and to propose a reimbursement system for this important part of hospital activity. For this purpose, we merged data contained in patient files with the results of monitoring the costs of the ED of E.O. Ospedali Galliera for a week. We found that although crude triage codes cannot be used to reimburse an ED, if they are combined with the number of laboratory and non-laboratory examinations, it is possible to cluster admissions to ED into three sufficiently homogeneous groups and each patient may be assigned to a specific group whose cost can be reimbursed using a PPS.

The PPS has been tested on our dataset. The results of a simulation suggest that our system may be robust enough to be used to reimburse for this activity. It shows a good ability to match reimbursement and actual cost incurred by the hospital.

Such a policy should discourage strategic behavior and allow the ED to break even, but one major limitation is that it may create an incentive for hospitals to increase the number of test/examinations to a fraction of green code patients to get a higher reimbursement.

## Endnote

^a^The 'internal price list’ provides the actual cost incurred by E.O. Ospedali Galliera in providing diagnostic tests or other services. The price is obtained by a bottom-up methodology by which all the costs that contribute to the overall production cost of that particular good or service are included in the computation of the final 'price’.

## Competing interests

Both authors declare that they have no competing interests.

## Authors’ contributions

RL and MM conceived and designed the study and they performed the statistical and economic analysis. Both authors approved the final version of the manuscript.

## Pre-publication history

The pre-publication history for this paper can be accessed here:

http://www.biomedcentral.com/1472-6963/13/409/prepub
